# Variables Affecting the Extraction of Antioxidants in Cold and Hot Brew Coffee: A Review

**DOI:** 10.3390/antiox13010029

**Published:** 2023-12-22

**Authors:** Brian G. Yust, Frank Wilkinson, Niny Z. Rao

**Affiliations:** 1 College of Humanities & Sciences, Thomas Jefferson University, Philadelphia, PA 19144, USA; 2 Department of Biological and Chemical Sciences, College of Life Sciences, Thomas Jefferson University, Philadelphia, PA 19144, USA; frank.wilkinson@jefferson.edu (F.W.); niny.rao@jefferson.edu (N.Z.R.)

**Keywords:** coffee, cold brew, caffeine, chlorogenic acid, antioxidants, extraction, nanoparticle, synthesis

## Abstract

Coffee beans are a readily available, abundant source of antioxidants used worldwide. With the increasing interest in and consumption of coffee beverages globally, research into the production, preparation, and chemical profile of coffee has also increased in recent years. A wide range of variables such as roasting temperature, coffee grind size, brewing temperature, and brewing duration can have a significant impact on the extractable antioxidant content of coffee products. While there is no single standard method for measuring all of the antioxidants found in coffee, multiple methods which introduce the coffee product to a target molecule or reagent can be used to deduce the overall radical scavenging capacity. In this article, we profile the effect that many of these variables have on the quantifiable concentration of antioxidants found in both cold and hot brew coffee samples. Most protocols for cold brew coffee involve an immersion or steeping method where the coffee grounds are in contact with water at or below room temperature for several hours. Generally, a higher brewing temperature or longer brewing time yielded greater antioxidant activity. Most studies also found that a lower degree of coffee bean roast yielded greater antioxidant activity.

## 1. Introduction

Coffee, one of the world’s most recognizable and readily available beverages, has continued to grow in both popularity and production over the past decades. Recently, total coffee bean production peaked at 176.6 million 60 kg bags in 2020/21 yet remains consistently high in the 165–175 million bag range [1]. The European Union and United States continue to drink the most coffee with domestic consumption around 40–42 million bags per year in the EU and 25–27 million bags per year in the US. While hot brewing techniques are still the most widely used, cold brew coffee has been growing in popularity [2]. In 2018, the global cold brew coffee market was valued at $339.7 million [3]. As one of the biggest new trends in coffee products, cold brew is forecast to have tremendous growth in the US market as well, swelling from $166 million in 2017 to an estimated $944 million by 2025 [4]. Beside the obvious difference in preparation, much is still not yet known about cold brew, and there are far fewer published research articles on cold brew coffee than hot brew methods. A few research groups have helped to bridge this gap by characterizing the chemical composition of cold brew coffee, including various antioxidants found in all coffee products. These antioxidants, usually extracted through a brewing process, can be a benefit to the health of coffee consumers or used for other chemical or industrial purposes.

Antioxidants from natural sources are an essential contributor to cellular and organismal health. Antioxidants are needed to neutralize reactive oxygen species (ROS) and other free radicals preventing chemical modifications to macromolecules and other metabolites. ROS arise from incomplete reduction of oxygen in the mitochondrial respiratory chain and other cellular enzyme systems [5]. Overproduction of ROS causes oxidative stress and can lead to cell death [6,7,8]. At high concentrations, ROS can cause chemical alterations to DNA, proteins, and lipids [9] and may lead to dysregulated gene expression or cancers [10]. Cells have mechanisms to combat ROS and lessen the damaging effects. Several enzymes serve as part of the antioxidative defense including superoxide dismutase, catalase, glutathione peroxidase, and glutathione reductase [11,12]. In addition to these systems, endogenous and environmentally sourced low molecular weight molecules can neutralize ROS and reduce oxidative stress [13]. Many compounds with antioxidant activity are present in plants and may be available to animal consumers such as those found in coffee. Because photosynthesis is the major source of molecular oxygen on Earth [14], organisms that utilize this pathway are vulnerable to oxidative stress due to the production of ROS supplementing that is produced in the mitochondria. Thus, the activity of plant-derived antioxidants which can act in hydrophilic and lipophilic environments provides added protection in addition to the enzymatic systems. 

People may acquire dietary antioxidants including vitamin C, vitamin E, and other bioactive molecules through a balanced, healthy diet [15]. Organisms which employ photosynthesis including plants are regularly exposed to high levels of oxygen, and the cellular defenses used to mitigate oxygen toxicity may be acquired by animal consumers to support their own physiological needs. In fact, diets including a high intake of plants, such as the paleolithic diet, may deliver significantly more antioxidants than modern diets [16]. While the connection between coffee consumption, in particular, and human health has been explored for many years, it is only recently that a generally positive relationship has been shown. For example, an earlier review of 22 studies in 1987 by Thelle et al. concluded that associations between serum cholesterol and coffee consumption were inconclusive [17]. Another study found that consumption of boiled coffee increased blood plasma cholesterol, which was attributed to the diterpenes, cafestol, and kahweol [18] even though diterpenes are usually removed in the process of filtering coffee [19,20]. One study which took genetic factors of the participants into account reported a U-shaped relationship between coffee consumption, cardiovascular disease, and mortality [21]. Other studies have suggested that coffee consumption has an inverse relationship with adverse health outcomes such as the occurrence of certain cancers [22,23,24,25,26,27], diabetes mellitus [28,29,30,31], depression [32,33,34], and even mortality [35,36,37,38].

Coffee products, both traditional and cold-brewed, have been found to contain many antioxidants which become bioavailable upon consumption [39]. The compounds responsible for these activities include phenolics (principally chlorogenic acids), caffeine, melanoidins, and volatile heterocyclic compounds [40,41,42,43,44]. Studies indicate that these activities can be partially absorbed across the intestine [45,46,47], though the persistence of these compounds in the gut can have potential in situ benefits for consumer and host microflora [48]. Beyond absorption and availability, these antioxidants have been found to increase the antioxidant capacity in the plasma of human subjects and for physiologic markers (i.e., reduced LDL oxidation) to be indicative of a protective effect [49,50,51]. Epidemiological evaluation of coffee consumers is consistent with the physiologic markers for reduced cardiovascular disease [52,53,54,55,56]. 

In general, a higher brewing temperature leads to greater antioxidant activity when other factors, such as brewing time, are constant [57,58]. The degree of roast is known to have an effect on the overall antioxidant activity as well. While many studies have found lower levels of antioxidant activity for darker roasts [59,60,61,62], there are some discrepancies between studies which used different methods to measure antioxidant activity, and it is unclear how to reconcile these findings. Longer brewing times will lead to higher antioxidant levels or activity up to a point, typically following a first-order kinetic model and plateauing after a few hours [59,63]. In cold brew, grind size does not have a significant impact on the final concentration of extracted molecules or antioxidant activity, likely due to the very long brewing times. In hot brew, contradictory findings have been reported on antioxidant concentration as a function of grind size [64,65,66,67,68]. Depending on the desired chemical or sensory profile, a brewing protocol can be optimized by altering the degree of roast, brewing temperature, and brewing time.

In addition to the human health benefits of the antioxidant activity of coffee, coffee-based products, extracts, and waste have been shown to be effective in synthesizing nanomaterials. The same redox reaction, which allows antioxidants to scavenge free radicals and ROS, can reduce nanoparticle precursors, such as metal ions, which promotes the aggregation and growth into nanoclusters [69,70,71,72]. This affords new green synthesis techniques by replacing hazardous and caustic precursors such as sodium borohydride, ammonium hydroxide, or hydrogen peroxide with a coffee-based extraction. For example, the antioxidant activity of phenolic acid and its ability to reduce Au (III) in particular has been studied and attributed to donation of electrons from the hydroxyl group [70]; meanwhile, electron donation from the oxygen in chlorogenic acid can stabilize the formation of Au nanoparticles [72,73,74]. Utilizing plant-derived antioxidants in nanoparticle synthesis has the added benefit of providing a secondary use for what might otherwise be food waste material, such as spent coffee grounds (SCG), as they are still rich in extractable antioxidants. SCG have been demonstrated to be as effective a reducing agent in nanoparticle synthesis as traditional reagents [75]. To date, coffee and coffee bean extract have been used to synthesize silver [75,76,77,78], gold [75,79], platinum [80], palladium [78], copper [81,82], zinc oxide [83], selenium [79], alumina [84], and carbon nanoparticles [85,86].

## 2. Antioxidant Activity in Coffee

The majority of extractable antioxidants, also referred to as antioxidant activity, in coffee beans, coffee products, and spent coffee grounds are due to naturally occurring polyphenols, primarily chlorogenic acids and their derivatives, and roasting induced melanoidins [87,88,89]. There are many different factors and variables which determine how and at what rate molecules are extracted from the coffee bean matrix. Some variables which stem from the growing conditions include the variety of the beans, regional soil conditions, and local weather and watering conditions. Other factors that arise from bean processing include the roasting temperature, roasting time, and grind size. Finally, even more variables are introduced in the production of the coffee product, such as the temperature of water during brewing, brewing duration, water to coffee ground ratio, and brewing method. Multiple studies have been published which characterize the antioxidant activity of coffee products. The difference in total antioxidant capacity between hot and cold brew coffees, though small, stands out as a detail worth noting (see below). When comparing the extraction of antioxidants in coffee to other plants, we see some commonalities. For example, extraction of antioxidants from mengkudu (*Morinda citrifolia*) was found to be dependent on time and temperature [90]. Phenolics, but not flavonoids, showed increased extraction and antioxidant activity over time. Phenolic extraction from coffee (using the filter brewing method) showed a U shaped extraction profile [41]. However, extraction of both phenolics and flavonoids increased with temperature [90]. Shang et al. found that pressurized liquid extraction recovered more antioxidant activity from black bamboo than reflux. The extracted antioxidants included phenolics and flavonoids and showed a 300% increase over low temperature extraction [91]. 

**Figure 1:**
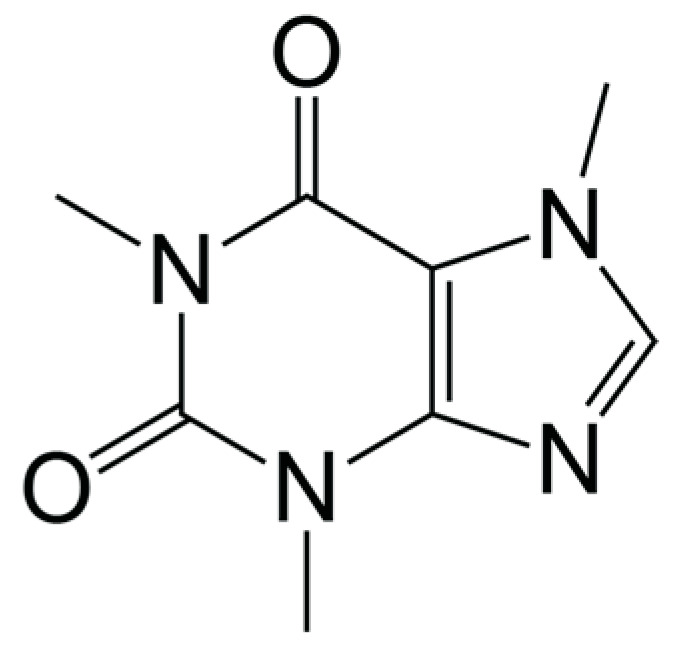
Molecular structure of caffeine.

Caffeine (Figure 1) itself also has been found to act as an antioxidant. In particular, caffeine was found to react with OH radicals through radical adduct formation and is predicted to be a modest scavenger of OCH_3_ and other alkoxyl radicals [92]. However, the same study showed that caffeine was not able to directly scavenge other ROS including OOCH_3_ and O_2_ radicals. This is in agreement with an earlier study which showed caffeine as highly effective in inhibiting lipid peroxidation from OH, moderately effective in inhibiting peroxidation by singlet molecular oxygen (^1^O_2_), and least effective in inhibiting peroxidation from peroxyl radicals (ROO) [93]. Another study used DNA encapsulated carbon nanotubes to compare the antioxidant potency of purified caffeine, hot drip coffee, decaffeinated hot drip coffee, and other antioxidants; their results indicate that while caffeine alone does scavenge peroxide and hydroxyl radicals, its potency as an antioxidant is weak when compared to coffee liquid, decaffeinated coffee, vitamin C, or uric acid [94].

## 3. Measuring Methods

**Figure 2:**
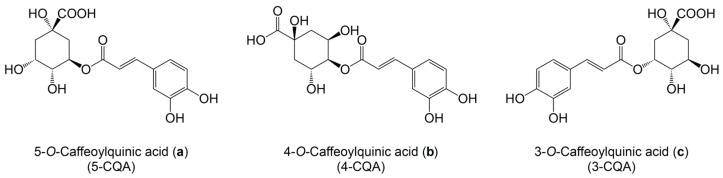
Molecular structures of three isomers of chlorogenic acids (CQA).

Numerous methods have been utilized in an effort to quantify extractable antioxidants in coffee and coffee products; however, there is no single standard methodology accepted across the industry. Each method measures some aspect of radical scavenging capacity by introducing the coffee product to a target molecule or reagent and then measuring a change in the electronic state or conformation, usually by spectroscopy or chromatography. Some of the commonly deployed systems for quantitative analysis of antioxidants in coffee include FRAP (ferric reducing antioxidant power), Trolox equivalence antioxidant capacity (TEAC), ABTS ((2,2′-Azino-bis(3-ethylbenzo-thiazonile-6-sulfonic acid) diammonium salt) radical scavenging), DPPH (2,2-diphenyl-1-picrylhydrazyl) radical scavenging, total antioxidant Capacity (TAC), determination of phenolic compounds by high-performance liquid chromatography (HPLC), and the Folin–Ciocalteu method for the determination of total phenolic content (TPC), among others [95]. A family of antioxidants found to be abundant in coffee, chlorogenic acids (CQA), are known to also play a significant role in the flavor profile of coffee products [96]. While these acids can contribute bitter and astringent flavors [96,97], they also have strong antioxidant activity [98,99,100]. Along with caffeine, the most commonly quantified antioxidants in coffee extracts are three isomers of chlorogenic acids (CGA), also commonly referred to as caffeoylquinic acids (CQA), 5-*O*-caffeoylquinic acid (Figure 2a) (5-CQA), 4-*O*-caffeoylquinic acid (Figure 2a,b) (4-CQA), and 3-*O*-caffeoylquinic acid (Figure 2c) (3-CQA), with 5-CQA being the major isomer of the CQA family. Historically, the nomenclature of CQAs have been inconsistent [98,101]. The early literature has used 3-CQA synonymously with 5-CQA. For the sake of conciseness, this review will focus on the reported concentration of the major isomer (3-CQA or 5-CQA) and the total CQA concentration. Finally, one can infer the degree of antioxidant extraction by examining what molecules are left behind in spent coffee grounds (SCG) which have already been brewed once by some method. These SCGs may be used to make a secondary, simple aqueous extract which can be analyzed through the same means as previously mentioned.

## 4. Coffee Beans

It has been demonstrated that the concentration of bioactive chemicals in coffee beans is influenced by their geographic origins [102,103,104,105,106,107,108]. The physicochemical profile of the coffee extract may also be influenced by additional variables, including growing circumstances [109], agricultural techniques [110,111], processing [112,113], storage [114], and genetic varietals [115]. Additionally, these factors also have significant influence on the flavor profile of the resulting brew [116]. There are two main coffee species, *Coffea arabica* and *Coffea canephora* var. Robusta, commonly referred to as Arabica and Robusta, respectively. There are some known differences in their chemical composition, physical bean characteristics, and sensory properties when brewed [117,118,119]. In general, coffee made with Robusta tends to have higher antioxidant activity and CQA concentrations while coffee made with Arabica tends to be richer in sucrose and oil [18,119]. However, a more detailed discussion is beyond the scope of this review. Most references cited here utilized *Coffea arabica* in their studies.

Within the specialty coffee market, peaberry coffee has recently gained popularity. Contrary to popular belief, peaberry coffee beans are not a genetic mutation. Rather, they result from the abortion of an ovary by a coffee plant leading to the fertilization and development of only one bean instead of two beans [120]. The primary physical difference between peaberry and regular coffee beans is the shape and size of the beans with peaberries appearing smaller and rounder compared to regular, flat-sided beans. Coffee producers have long argued that peaberry coffee would have better flavor than regular coffee as the nutrients are delivered to one bean only [121]. To date, research on the peaberry coffee bean is scarce, largely due to limited availability as roughly 7% of harvested farm crops are identified as peaberry beans [122,123]. A recent study by Schwarzmann et al. showed that the extracted antioxidants in peaberry coffee from various regions did not vary with brewing temperature [124]. The authors analyzed the total antioxidant capacities of cold and hot brew extracts of peaberry coffee from four regions. They found that total antioxidant capacity (TAC) values of peaberry coffee extracts, regardless of brewing temperature, did not correlate to either caffeine concentration or CQA concentration, indicating that neither compound was a major contributor to the antioxidant activities of peaberry coffee extract. Additional research is urgently needed to fully elucidate the difference between regular and peaberry coffee.

## 5. Brewing Temperature

Whether coffee is made by a hot brew, cold brew, or espresso method, it will involve an aqueous extraction process whereby various compounds diffuse out of the ground beans and into the water, imbuing it with the characteristic taste, aroma, and color of coffee. Extraction of compounds from the beans will depend on the water temperature, volume, and duration of contact [57]. Additionally, loss of volatile aromatic compounds due to evaporation is less prominent for cold brew than for high-temperature extraction.

A study by Stanek et al. found that the antioxidant activity as measured by ABTS and DPPH was higher in hot-brewed samples compared to cold-brewed samples for coffee prepared from medium-dark roast beans sourced from four different regions (see Table 1) [58]. Interestingly, this work also demonstrated that a new cold-brew technique using coffee bed percolation, referred to as Hardtank, increased the antioxidant activity of cold-brew, making them similar to hot coffee samples. Another study found that brewing medium roast beans at a higher temperature resulted in significantly greater total phenolic content (TPC) as measured by FC assay and greater antioxidant activity as measured by ABTS [125]. These trends held for drip, pour over, and steeping methods. A similar study examined the time evolution of phenolics and antioxidants extracted from medium roast beans, showing that there is a significant increase of both in cold brewed coffee, especially in the 6–12 h brew-time window [126]. Xu et al. demonstrated that antioxidant activity measured as ABTS scavenging activity increased directly with temperature but followed a biphasic curve with time (maximum at 45–50 min). Meanwhile, DPPH scavenging activity was largely constant with temperature but declined significantly with time [127]. The same study found that phenolics extracted from spent coffee grounds increased with both time and temperature [127]. A detailed study by Pan et al. which investigated the influence of brewing temperature and degree of roast on the chemical profile of coffee found that hot brew consistently had higher antioxidant activity than cold brew as measured by DPPH, ABTS, and TPC (see Table 1) [128]. Their study also found higher caffeine concentrations in cold brew but did not note any significant difference in CQA concentration between hot and cold brew samples. One published report investigated the difference between hot brew, cold brew at room temperature, and cold brew at 4 °C, finding that the caffeine and CQA concentrations for room temperature cold brew and hot brew were quite similar, but significantly lower for the cold brew prepared at 4 °C [129]. The same work also showed both cold brew types had higher pH and lower titratable acid than the hot brew counterparts, explaining a lower perceived acidity of cold brew.

## 6. Roasting Temperature

The high temperatures used in roasting coffee beans have a significant impact on the extractable chemicals. For example, total CQA concentration is known to decrease for coffee beans roasted at higher temperatures. In general, roasting decreases the amount of CQA in coffee beans through chemical transformation [60,130]. Factors which affect the extractability and structure of the CQA will obviously have an effect on both the flavor and direct health benefits. Using mass spectroscopy, the reaction of CQA during roasting were studied and found to follow one of four reaction schemes, including acyl migration, dehydration, epimerization, and lactonization [131]. Unsurprisingly, roast level has been found to affect CQA concentration in both hot and cold brew coffee. For example, it was reported by Fuller and Rao [59] that the 5-CQA concentration in Kona coffee was 485 ± 47 mg/L for medium roast, significantly higher than the 355 ± 51 mg/L for dark roast after 400 min of cold brewing (see Table 1), consistent with previous studies on hot brewed coffee [61,130,132,133,134,135]. In a follow up study, they found a general inverse trend between 5-CQA concentration and degree of roast in Colombian coffee with 757 ± 27 mg/L, 353 ± 15 mg/L, and 147 ± 14 mg/L reported for light, medium, and dark roasts, respectively, after 420 min of brewing [136]. These marked differences in the extracted CQA concentrations will affect both the overall antioxidant activity as well as the flavor profiles of the coffee product.

The total antioxidant capacity (TAC) of coffee is thought to be a good way to generally understand the total effect that a complex and varied mixture of antioxidants such as coffee can have without attributing the antioxidant activity to any specific constituents. However, the specific ways in which each TAC measurement method interacts with specific compounds has led to some discrepancies between reported studies with similar experimental conditions. As an example, TAC was reported to decrease as the degree of roast increased for cold brew coffee when measured by ABTS radical cation decolorization assay [105] and 2,2-diphenyl-1-picrylhydrazyl (DPPH) decolorization assay [106]. However, Bilge also noted that the total phenolic content (TPC) did not exhibit any correlation with the degree of roast. Such discrepancies have been well documented for hot brew coffee as well [40,43,137,138,139,140,141,142,143,144]. Schouten et al. found that the initial roasting in light-roast coffee beans greatly increased the antioxidant activity of the coffee as measured by FRAP, FC, ABTS, and DPPH assays, when prepared by a hot-brew method [62]. As the degree of roast continued into medium-roast, the antioxidant activity peaked and then slightly decreased more so for dark-roast samples. More recently, Pan et al. found that antioxidant activity as measured by DPPH, ABTS, and TPC for both hot and cold brew decreased as the degree of roast went from light to medium and dark (see Table 1) [128]. This also held for coffee made from beans sourced from two separate growing regions.

## 7. Brewing Time

The amount of contact time between the coffee grounds and water will have a large impact on the final chemical profile of coffee. Espresso, French press, percolation, drip, and pour-over brew methods occur on the order of seconds to minutes. In contrast, cold brew methods tend to take hours. It has been shown that in hot brew coffee, highly soluble compounds including caffeine, sugars, and organic acids are extracted within the first few seconds of brewing [145]. Meanwhile, less soluble compounds, such as chlorogenic acid lactones and other bitter compounds, are only extracted either through longer contact time between the grounds and water or by using a greater volume of water during the brewing process [146,147]. At lower brewing temperatures, the solubility of many compounds decreases significantly. Low polarity compounds do not extract as efficiently at low temperatures and therefore require a longer brewing time to reach a desired concentration range [147]. 

Since cold brewing has increased in popularity, more studies have been published on the characteristics of these products. Most reported methods are carried out at or near room temperature (20–25 °C or colder) with the ground coffee being steeped for 6–24 h. Most hot-brewing techniques, by contrast, use a much shorter contact time on the order of a few minutes with an optimal temperature around 93 °C [148]. Polar, soluble compounds such as furans, ketones, acids, and sugar are known to be readily solubilized at lower temperature, while less polar compounds often associated with bitterness require higher temperatures to be extracted from the coffee bean matrix [147]. The longer brewing times used for cold brew coffee likely affect the final coffee composition since the diffusion of molecules through the matrix of the coffee grounds may be kinetically limited. The complex interactions between the solid bean-based matrix and the water can vary over the duration of brewing, and it has been shown that faster chemical extraction occurs at or near the surface of the coffee granules [149,150]. With longer brewing times, cold brewing methods may be able to extract compounds found deeper inside the coffee bean matrix.

A study by Kim and Kim first reported that both caffeine and CQA concentrations varied by extraction time for cold-brewed coffee. They compared the drip method which demonstrated an inverse relationship between extraction time and caffeine or CQA concentrations to the steeping method which demonstrated a direct correlation between extraction time and caffeine or CQA (see Table 2). Another finding was that the sensory profile of cold brew coffee made by the drip method with a total extraction time of 18 h was preferable to coffees brewed with shorter extraction times [151]. Conversely, Angeloni et al. did not find any strong correlation between the extraction time of cold brewed coffee and its chemical or sensory profile [152]. Antioxidant levels and total antioxidant capacities (TAC) of cold brew coffee have also been found to depend on the total extraction time. The extraction of 5-CQA by cold-brewing was reported to follow a first-order kinetic model, rapidly increasing during the first 180 min and then plateauing to a steady–steady state concentration after approximately 400 min [59]. Maksimowski et al. recently found little correlation between extraction time and either caffeine or chlorogenic acid concentrations in cold brew after 6, 12, and 24 h [63]. While their exact concentrations and analytic techniques differ from those reported by Rao and Fuller, there is agreement that the first few hours of extraction time seem to have a greater effect on these overall concentrations. Han et al. observed that both total phenolic compounds (TPC) and TAC also increased with longer extraction times [126]. Another recent study compared espresso, French press, cold brew, and AeroPress prepared samples [128]. Espresso and cold brew were found to have more effective extraction methods, particularly with regard to their total polyphenolic content (TPC), caffeine concentration, and CQA concentration. While the temperature and pressure used in the espresso samples can explain the reported higher levels, the longer brewing time used in cold brew samples is attributed to their higher levels when compared to French press and AeroPress prepared samples. Additionally, the espresso and cold brew samples from the same study also showed higher values of antioxidant activity as measured by DPPH and TPC. In general, longer extraction times correlate to higher antioxidant concentrations in cold brew coffees [59,126]. However, when accounting for taste, the sensory profile of cold brew coffee with shorter extraction times were favored for their reduced bitterness, astringency, and aftertaste [107,126].

When examining the kinetics of caffeine extraction, mathematical models found that diffusion of caffeine at room temperature from the intragranular pore space, defined as the pore space within each grain of coffee, to the intergranular network, or space between grains of coffee, was the rate limiting step in the overall extraction process [149,150,153]. Due to this, the time needed to reach equilibrium in the extraction process increases with grind size, even at higher extraction temperatures. Because cold brew processes utilize such long extraction time scales, in the order of hours, the slow diffusion from intragranular pores to intergranular space is not rate-limiting in cold brew methods. Therefore, concentrations of caffeine tend to be higher in cold brew coffee prepared using longer extraction times [59,126] and smaller grind particle sizes [107].

## 8. Brewing Method (Immersion vs. Drip)

When comparing brewing methods broadly, immersion and drip methods exhibit a significant difference in the final caffeine concentration of a coffee product. This can be attributed to the more dynamic milieu of a drip method where fresh water is added continuously to the brewing chamber, resulting in a concentration gradient in the liquid which is largely responsible for mass transfer of soluble compounds from the coffee bean matrix into the water [154]. In a steeping process, the concentration gradient is nearly uniform since the entire volume of liquid is in contact with the coffee grounds throughout the brewing process. A consequence of this difference is that the drip method tends to be more efficient at extracting compounds into the coffee liquid than the steeping method. This can be seen, in particular, when comparing caffeine concentrations as in cold brew coffee samples listed in Table 3 [151,152].

However, other antioxidants in cold brew coffee exhibited little variation between brewing methods. Various groups have reported that 5-chlorogenic acid concentration (5-CQA) [152], total phenolic content (TPC) [125,126], and antioxidant activity (AA) [125,126] were not significantly different between the two brewing methods (see Table 4). 

## 9. Grind Size

Particle size of the coffee grounds is thought to influence the extraction rates of chemicals from the coffee bean matrix into water during the brewing process, influencing both the flavor and chemical profile of the final coffee product. One study showed that coffee bed density, which arises from differential particle size, plays a critical role in bed permeability, thereby directly affecting coffee extraction [156]. Mathematical modeling of chemical extraction from the coffee bean matrix proposed by Moroney et al. [150,153] suggests that diffusion from the intragranular pores to the intergranular pores is the rate limiting process. This would lead to a shorter time for the extraction process to reach equilibrium as the grind size decreases. In the case of cold brew coffee, the extraction time frame is in the order of hours instead of seconds, differentiating it from hot brew processes. As such, Rao and Fuller found that the grind size does not have a significant impact on the extracted concentrations for both CQA and caffeine in cold brew coffee. Such long extraction time scales allow for the slow diffusion from intragranular to intergranular pores with concentrations of CQA and caffeine reaching equilibrium at about 400 min. In hot brew coffee, caffeine concentration has been reported to have a direct relationship to grind size with more coarse coffee yielding higher concentrations by one group [68] and an inverse relationship to grind size with finer coffee yielding higher caffeine concentrations when brewing method and contact time were held constant across samples by other groups [64,65,66,67].

## 10. Indirect Assessment of Antioxidant Extraction through Spent Coffee Grounds

Many previous studies have found that, despite having undergone an initial brewing process, spent coffee grounds (SCGs) may retain high concentrations of various molecules [157,158,159,160,161,162,163,164,165]. Residual antioxidant activities have also been demonstrated to be available in SCGs [88,162,166]. As with measuring antioxidant levels and activity in a first brew of coffee, the methods used to measure the same aspects of SCGs may use different extraction techniques which can involve aqueous or methanolic solutions, microwave assistance, or a variety of other means. A few works have elucidated how various brewing methods [160,165,167] or degree of roast [160,168] might affect what compounds are left behind in SCGs. Yust et al. recently analyzed the total CQA concentrations and TAC of extracts from six SCG samples generated from medium and dark roast beans by various brewing methods [75]. Since these values reflect what extractable compounds remain after the initial brewing process, one can infer the concentration of molecules present in the initial coffee product. Therefore, SCG samples with a low value indicate that the initial coffee product would have a high concentration and vice versa. From the data in Table 5, it can be seen that an initial hot French press brewing method did not extract CQAs as efficiently as a cold brew method or espresso brewing, therefore leaving significant CQAs behind in the SCGs. Espresso-derived SCG extracts exhibiting low levels of CQA concentration are in agreement with a previous study by Bravo et al. [165] and corroborates the high extraction efficiency of the espresso brewing method in comparison to other brewing methods [41,118,169]. Andrade et al. also quantified the antioxidant activity of espresso SCGs from beans grown in different regions, finding that SCGs from Brazil and Ethiopia had scavenged more radicals than SCGs from Guatemala or Colombia when measured using DPPH [170]. However, when measured using ABTS, SCGs from Ethiopia scavenged more radicals than the other SCGs.

The extraction of antioxidant activities from coffee grounds can have impacts beyond the beverage itself. Recently, a group used SCG with thermoplastic starch as a component of a bioplastic blend [171]. Blends with 15% and 20% SCG showed radical scavenging activity. Although the extraction of antioxidants from coffee grounds was not quantitative in the methods used to prepare the SCG, the amount of antioxidant activity available for downstream applications is predicted to be influenced by the extent of extraction during upstream processing. There has also been interest in exploring new technologies for extracting the remaining antioxidants from SCG including microwave heating [172,173], ultrasonic agitation [174,175], supercritical fluid extraction such as CO_2_ [176,177], and subcritical fluid extraction such as water [127,178]. Utilizing coffee byproducts such as SCG as a resource from which to extract useful compounds including antioxidants can further the goals of reducing waste, supplementing more harmful sources and processes for producing similar compounds, and offering a new revenue stream for coffee processors and producers. 

## 11. Conclusions

Coffee is a culturally and economically important part of our modern world. As coffee has gained popularity globally, so has its availability. Coffee beans and coffee products are an abundant source of antioxidants which can be beneficial to people when consumed or alternatively used in some other capacity such as nanoparticle synthesis. The most abundant antioxidants found in coffee extracts include chlorogenic acids, derivatives of chlorogenic acid, other polyphenols, melanoidins, and caffeine. There are a multitude of variables which affect the extraction of antioxidants including coffee bean varietal, growth region and conditions, brewing temperature, brewing duration, roasting temperature, and grind size. At low brewing temperature, the antioxidant activities of cold brew coffee are particularly sensitive to variation in the aforementioned attributes. A better understanding of the physicochemical profiles of cold brew coffee extracts not only will aid the development of new and innovative brewing methods to meet the demand of coffee consumers worldwide but will also guide future development in coffee waste recycling. Additionally, climate change is having a pronounced effect on growing conditions such as annual rainfall, average and peak temperatures, and severe flooding around the globe, and a better understanding of how these changes will influence the chemical profile of coffee is still needed. There has been a noticeable increase in published research concerning cold brew techniques and products in recent years, yet a greater understanding of the intricacies involved is still possible. In general, higher temperature brewing leads to a quicker extraction of antioxidant compounds from the coffee bean matrix than lower temperature brewing. However, brewing at a higher temperature for too long can lead to a more bitter flavor profile; this is one of the reasons for shorter typical contact time for these methods. Longer brewing time can lead to a more thorough extraction of coffee bean extracts, such as in most cold brewing protocols. The degree of coffee bean roast also has a significant impact on the extractable antioxidants, with darker roasts exhibiting a lower levels of antioxidant activity due to the decomposition of antioxidants during the roasting process. The grind size does not seem to have a particularly strong role to play in antioxidant extraction for cold brew, perhaps due to the long brewing times used. For hot brewing techniques, some groups found an inverse relationship between grind size and caffeine concentrations, but a direct relationship has also been reported. For the coffee consumer, perceived acidity and sweetness in coffee tend to be more pronounced with higher antioxidant concentrations, particularly chlorogenic acids. Meanwhile, astringency, bitterness, and aftertaste tend to be less noticeable with higher antioxidant activity. Spent coffee grounds (SCGs) also have copious amounts of antioxidants left over after the initial brewing process. They can, therefore, be used as a way to infer how effective the antioxidant extraction was. SCGs that were quantified showed that French press SCG have the highest amount and espresso SCG have the least amount of extractable antioxidants. This can also inform potential applications for coffee waste. Finally, the variables considered herein may be tailored for a cold or hot brewing technique to yield a desired chemical profile or antioxidant activity of a coffee product.

## Figures and Tables

**Table 1:** Select antioxidant measurements in coffee of various origin, degree of roast, and brew temperature.

Bean Origin	Degree of Roast	Brew Temperature	Antioxidant Activity (DPPH Assay, mg TE/g)	Antioxidant Activity (ABTS Assay mg TE/g)	Total Phenolic Content (TPC, mg GAE/g)	Total Flavonoid Content (TFC, mg QE/g)	5-CQA (mg/100 g)	Caffeine (mg/100 g)	Total Antioxidant Capacity (mmol Trolox/L)	Reference
Guatemala, *Coffea Arabica*	Medium-Dark	19.3 °C	34.99 ± 2.53	33.73 ± 0.71	25.92 ± 1.87	3.07 ± 0.13	260 ± 2	711 ± 9		Stanek [58]
		96 °C	45.53 ± 0.34	51.14 ± 1.56	33.46 ± 1.10	3.67 ± 0.06	279 ± 2	770 ± 3		
El Salvador, *Coffea Arabica*	Medium-Dark	19.3 °C	27.63 ± 2.88	39.80 ± 0.33	21.50 ± 0.68	3.04 ± 0.05	227 ± 1	668 ± 17		Stanek [58]
		96 °C	32.61 ± 1.14	39.80 ± 0.33	23.04 ± 0.89	3.70 ± 0.11	229 ± 5	705 ± 17		
Brazil, *Coffea Arabica*	Medium-Dark	19.3 °C	39.08 ± 1.20	37.86 ± 0.53	23.46 ± 0.44	3.11 ± 0.22	261 ± 17	705 ± 44		Stanek [58]
		96 °C	40.03 ± 1.74	49.17 ± 3.02	23.43 ± 0.62	3.44 ± 0.19	234 ± 15	689 ± 40		
Bolivia, *Coffea Arabica*	Medium-Dark	19.3 °C	42.87 ± 1.02	54.68 ± 0.49	23.77 ± 0.61	3.18 ± 0.12	280 ± 3	605 ± 13		Stanek [58]
		96 °C	39.07 ± 1.47	55.02 ± 2.77	26.90 ± 1.31	3.39 ± 0.23	263 ± 13	601 ± 24		
Uganda, *Coffea Arabica*	Medium	5 °C		3249.31 ± 287.82 mg VcE/L	9 μmol/mL GAE ^a^					Kang [125]
		20 °C		4243.40 ± 290.46 mg VcE/L	10.91 ± 0.48 μmol/mL GAE					
		95 °C		3604.00 ± 354.61 mg VcE/L	7.5 μmol/mL GAE ^a^					
Uganda, *Coffea Arabica*	Medium	5 °C		1075 mg VcE/L ^a^	9 μmol/mL GAE ^a^					Han [126]
		10 °C		1100 ish mg VcE/L ^a^	9 μmol/mL GAE ^a^					
		20 °C		1175 mg VcE/L ^a^	11.5 ish μmol/mL GAE ^a^					
Kona, *Coffea Arabica, Kona Typica*	Medium	21–25 °C					485 ± 47 mg/L	1095 ± 55 mg/L		Fuller [59]
	Dark	21–25 °C					355 ± 51 mg/L	950 ± 51 mg/L		
Colombia, *Coffea Arabica*	Light	21–25 °C					757 ± 27 mg/L	1114 ± 56 mg/L	13.09 ± 0.22	Rao [105]
	Medium	21–25 °C					353 ± 15 mg/L	1036 ± 19 mg/L	11.11 ± 0.33	
	Dark	21–25 °C					147 ± 14 mg/L	962 ± 41 mg/L	10.13 ± 0.59	
Sumatra, *Coffea Arabica*	Light	5 °C	6.75 ± 0.12 mmol Trolox/L	2.14 ± 0.07 mmol Trolox/L	0.83 ± 0.03 mg/mL					Pan [128]
	Medium		6.01 ± 0.11 mmol Trolox/L	1.74 ± 0.09 mmol Trolox/L	0.68 ± 0.08 mg/mL					
	Dark		4.03 ± 0.13 mmol Trolox/L	1.44 ± 0.08 mmol Trolox/L	0.58 ± 0.02 mg/mL					
	Light	92 °C	7.61 ± 0.17 mmol Trolox/L	2.25 ± 0.05 mmol Trolox/L	0.95 ± 0.05 mg/mL					
	Medium		6.89 ± 0.12 mmol Trolox/L	2.03 ± 0.07 mmol Trolox/L	0.80 ± 0.03 mg/mL					
	Dark		5.54 ± 0.14 mmol Trolox/L	1.62 ± 0.07 mmol Trolox/L	0.67 ± 0.03 mg/mL					
Yunnan, *Coffea Arabica*	Light	5 °C	6.41 ± 0.15 mmol Trolox/L	1.93 ± 0.03 mmol Trolox/L	0.75 ± 0.03 mg/mL					Pan [128]
	Medium		5.66 ± 0.12 mmol Trolox/L	1.57 ± 0.06 mmol Trolox/L	0.66 ± 0.02 mg/mL					
	Dark		3.13 ± 0.23 mmol Trolox/L	1.08 ± 0.05 mmol Trolox/L	0.51 ± 0.03 mg/mL					
	Light	92 °C	7.22 ± 0.12 mmol Trolox/L	2.23 ± 0.08 mmol Trolox/L	0.86 ± 0.02 mg/mL					
	Medium		6.45 ± 0.12 mmol Trolox/L	1.88 ± 0.06 mmol Trolox/L	0.81 ± 0.04 mg/mL					
	Dark		5.33 ± 0.21 mmol Trolox/L	1.32 ± 0.11 mmol Trolox/L	0.58 ± 0.07 mg/mL					

^a^ Values estimated from figures included in the referenced papers.

**Table 2:** Select antioxidant measurements in coffee of various origin, degree of roast, and brew temperature as a function of brewing time.

						Brewing Duration								
Measurement	Bean Origin	Degree of Roast	Brew Method	Brew Temperature	3 min	15 min	1 h	3 h	6 h	9 h	12 h	18 h	24 h	Reference
3-CQA (mg/L)	Kona, *Coffea Arabica*	Medium	Steep	21–25 °C		64.9 ± 46.1	164.7 ± 89.9	319.5 ± 66	442.3 ± 72	479.6 ± 40	475.1 ± 47		508.8 ± 29.8	Fuller [59]
		Dark	Steep	21–25 °C		83.3 ± 37.7	148.8 ± 12.4	271.4 ± 48.8	302.1 ± 73.5	361.9 ± 17.6	410.3 ± 33.6		393.8 ± 67.8	
CQA (ppm)	Kenya, *Coffea Arabica*	Dark	Drip	21–25 °C				2122.54 ± 194.98	1461.33 ± 203.56	1176.12 ± 70.37		612.23 ± 12.61		Kim [151]
			Steep	21–25 °C				193.94 ± 14.06	233.16 ± 32.74	255.26 ± 29.39		291.05 ± 40.77		
CQA (mg/L)	Brazil, *Coffea Arabica*	Light	Steep	5 °C					1036.2 ± 22.5		902.5 ± 12.5		949.2 ± 16.3	Maksimowski [63]
				10 °C					1034.5 ± 23.2		930.5 ± 29.4		964.0 ± 11.6	
				15 °C					919.4 ± 4.7		931.9 ± 6.9		921.4 ± 29.4	
Caffeine (mg/L)	Kona, *Coffea Arabica*	Medium	Steep	21–25 °C		158.3 ± 114.7	390.2 ± 172.9	730 ± 123.5	983.4 ± 62	1077 ± 82.8	1071.7 ± 61.3		1182.9 ± 124.2	Fuller [59]
		Dark	Steep	21–25 °C		156.4 ± 63.1	440.2 ± 45.4	776 ± 96.5	805.9 ± 101.4	991.8 ± 38.1	1124.7 ± 92.8		1075.8 ± 91.4	
Caffeine (ppm)	Kenya, *Coffea Arabica*	Dark	Drip	21–25 °C				5288.99 ± 480.58	3818.39 ± 503.48	3139.42 ± 166.88		1606.05 ± 30.98		Kim [151]
			Steep	21–25 °C				509.99 ± 44.64	619.39 ± 100.88	685.13 ± 63.65		755.64 ± 106.47		
Caffeine (mg/L)	Brazil, *Coffea Arabica*	Light	Steep	5 °C					460.2 ± 8.1		474.6 ± 10.6		540.4 ± 24.3	Maksimowski [63]
				15 °C					516.1 ± 11.7		526.0 ± 26.2		497.4 ± 10.8	
				25 °C					471.2 ± 9.4		473.5 ± 6.5		500.9 ± 9.9	
Total Phenolic Content (TPC, μmol GAE/mL)	Uganda, *Coffea Arabica*	Medium	Drip	5 °C							6.75 ^a^			Kang [125]
				20 °C							10.75 ^a^			
			Pour Over	80 °C	7 ^a^									
				95 °C	7.75 ^a^									
			Steep	5 °C							8.75 ^a^			
				20 °C							11 ^a^			
Total Phenolic Content (TPC, μmol GAE/mL	Uganda, *Coffea Arabica*	Medium	Steep	5 °C				6 ^a^	7.5 ^a^		8.75 ^a^		9 ^a^	Han [126]
				10 °C				7 ^a^	7.5 ^a^		8.75 ^a^		9 ^a^	
				20 °C				7.5 ^a^	8.75 ^a^		11.25 ^a^		11.5 ^a^	
			Drip	10 °C				6.75 ^a^	7.5 ^a^		8.5 ^a^			
				20 °C				7.5 ^a^	8.5 ^a^		10.5 ^a^			
Antioxidant Activity (AA-ABTS assay mg VcE/L)	Uganda, *Coffea Arabica*	Medium	Drip	5 °C							3250 ^a^			Kang [125]
				20 °C							4400 ^a^			
			Pour Over	80 °C	3300 ^a^									
				95 °C	3600 ^a^									
			Steep	5 °C							3250 ^a^			
				20 °C							4300 ^a^			
Antioxidant Activity (AA-ABTS assay mg VcE/L)	Uganda, *Coffea Arabica*	Medium	Steep	5 °C				550 ^a^	775 ^a^		1000 ^a^		1075 ^a^	Han [126]
				10 °C				575 ^a^	800 ^a^		1050 ^a^		1100 ^a^	
				20 °C				650 ^a^	850 ^a^		1100 ^a^		1175 ^a^	
			Drip	10 °C				550 ^a^	750 ^a^		1000 ^a^			
				20 °C				600 ^a^	800 ^a^		1050 ^a^			

^a^ values estimated from figures included in the referenced papers.

**Table 3:** Average caffeine concentration of cold brew coffee as a function of extraction methods.

Drip	Steeping	Reference
1.03 ± 0.19 mg/mL	0.85 ± 0.15 mg/mL	Angeloni [152]
3.46 ± 0.30 mg/mL	0.64 ± 0.08 mg/mL	Kim [151]
1.42 ± 0.008 mg/mL	0.995 ± 0.005 mg/mL	Cordoba [155]
1.33 ± 0.009 mg/mL	0.944 ± 0.005 mg/mL	Cordoba [155]

**Table 4:** Select chemical attributes of cold brew coffee as a function of extraction method.

Compound	Drip	Steeping	Reference
5-CQA	0.36 ± 0.07 mg/mL	0.29 ± 0.06 mg/mL	Angeloni [152]
TPC ^a,b^	10.76 ± 0.49 µmol/mL GAE	10.91 ± 0.48 µmol/mL GAE	Kang [125]
TPC ^a,c^	10.2 µmol/mL GAE	10.8 µmol/mL GAE	Han [126]
AA ^d^	3747.36 ± 289.1 mg VcE/L	3817.61 ± 297.6 mg/VcE/L	Kang [125]
AA ^d,c^	1000 mg VcE/L	1100 mg VcE/L	Han [126]

^a^ TPC values were reported as µmol gallic acid equivalent (GAE) per mL of coffee brew. ^b^ reported value were measured at 20 °C by Kang et al. [125]. ^c^ values estimated for extraction at 20 °C for 12 h from figures by Han et al. [126]. ^d^ AA values were reported as mg vitamin C equivalent (VcE) per liter of coffee brew.

**Table 5:** Antioxidant measurements of spent coffee ground (SCG) extracts.

Origin, Degree of Roast	Initial Brewing Method	Total CQA Concentration (mg/L of Extract)	ABTS (mmol TE/L Extract)	DPPH (mmol TE/L Extract)	TPC (mg GAE/L Extract)	FRAP (mg FeSO_4_/L Extract)	Reference
Colombia, *Coffea Arabica* Medium	Cold, Immersion	480.65 ± 8.31 ^a,A^	5.69 ± 0.59 ^a,A^	4.23 ± 0.68 ^a,A^	420.5 ± 16.7 ^a,A^	145.6 ± 4.2 ^a,A^	Yust [75]
	Hot, French Press	716.02 ± 7.70 ^b,A^	6.94 ± 0.64 ^b,A^	5.69 ± 1.54 ^ab,A^	534.2 ± 14.0 ^b,A^	217.1 ± 12.3 ^b,A^	
	Hot, Espresso	221.12 ± 1.17 ^c,A^	5.5 ± 0.37 ^b,A^	3.57 ± 0.72 ^b,A^	313.5 ± 8.9 ^c,A^	111.4 ± 6.8 ^c,A^	
Colombia, *Coffea Arabica* Dark	Cold, Immersion	202.72 ± 3.67 ^a,B^	7.15 ± 0.78 ^a,B^	5.41 ± 1.05 ^a,B^	503.2 ± 12.6 ^a,B^	193.3 ± 6.9 ^a,B^	Yust [75]
	Hot, French Press	277.86 ± 4.47 ^b,B^	8.92 ± 0.59 ^b,B^	7.6 ± 0.79 ^b,A^	595.2 ± 11.0 ^b,B^	254.6 ± 16.6 ^b,B^	
	Hot, Espresso	38.88 ± 0.71 ^c,B^	3.43 ± 0.20 ^c,B^	2.53 ± 0.39 ^c,A^	206.2 ± 21.1 ^c^	73.1 ± 2.7 ^c,B^	
Colombia, *Coffea Arabica*	Hot, Espresso		0.75 mg TE/100 g DW ^+^	52 mg TE/100 g DW ^+^	41.6 ± 2.1 mg GAE/100 g DW		Andrade [170]
Brazil, *Coffea Arabica*	Hot, Espresso		0.85 mg TE/100 g DW ^+^	78.1 ± 7.3 TE/100 g DW	53.7 ± 3.1 mg GAE/100 g DW		Andrade [170]
Guatemala, *Coffea Arabica*	Hot, Espresso		0.5 ± 0.04 mg TE/100 g DW	50.6 ± 5.3 mg TE/100 g DW	52 mg GAE/100 g DW ^+^	50.6 ± 5.3 mg TE/100 g DW	Andrade [170]
Ethiopia, *Coffea Arabica*	Hot, Espresso		1.8 ± 0.2 mg TE/100 g DW	66 mg TE/100 g DW ^+^	42 mg GAE/100 g DW ^+^		Andrade [170]

Values are reported as mean ± SD with *n* = 6. The superscripts a–c denote significant (*p* < 0.05) differences among brewing methods at the same degree of roast as determined by the Tukey HDS post-tests. The superscripts A and B denote significant differences between medium and dark roast within the same brewing method. ^+^ Other values estimated from figures included in the referenced papers.
